# Expression of Main Toll-Like Receptors in Patients with Different Types of Colorectal Polyps and Their Relationship with Gut Microbiota

**DOI:** 10.3390/ijms21238968

**Published:** 2020-11-26

**Authors:** Sama Rezasoltani, Reza Ghanbari, Mehdi Azizmohammad Looha, Ehsan Nazemalhosseini Mojarad, Abbas Yadegar, Delisha Stewart, Hamid Asadzadeh Aghdaei, Mohammad Reza Zali

**Affiliations:** 1 Basic and Molecular Epidemiology of Gastrointestinal Disorders Research Center, Research Institute for Gastroenterology and Liver Diseases, Shahid Beheshti University of Medical Sciences, Tehran 1985717411, Iran; samasoltani70@gmail.com (S.R.); mehdi.looha@gmail.com (M.A.L.); 2 Digestive Oncology Research Center, Digestive Diseases Research Institute, Tehran University of Medical Sciences, Tehran 1411713135, Iran; r.ghanbari98@gmail.com; 3 Gastroenterology and Liver Diseases Research Center, Research Institute for Gastroenterology and Liver Diseases, Shahid Beheshti University of Medical Sciences, Tehran 1985717411, Iran; ehsanmojarad@gmail.com (E.N.M.); nnzali@hotmail.com (M.R.Z.); 4 Foodborne and Waterborne Diseases Research Center, Research Institute for Gastroenterology and Liver Diseases, Shahid Beheshti University of Medical Sciences, Tehran 1985717411, Iran; a.yadegar@sbmu.ac.ir; 5 Nutrition Research Institute, University of North Carolina at Chapel Hill, Kannapolis, NC 28081, USA

**Keywords:** pattern recognition receptors, Toll-like receptors, colorectal polyps, gut bacteria

## Abstract

Abnormal activation of Toll-like receptor (TLRs) signaling can result in colon cancer development. The aim of this study was to investigate the expression of important TLRs in different histological types of colorectal polyps and evaluate their relationship with intestinal microbiota. The expression levels of TLR2, 3, 4, and 5 were analyzed in intestinal biopsy specimens of 21 hyperplastic polyp (HP), 16 sessile serrated adenoma (SSA), 29 tubular adenoma (TA), 21 villous/tubulovillous (VP/TVP) cases, and 31 normal controls. In addition, selected gut bacteria including *Streptococcus bovis*, *Enterococcus faecalis*, Enterotoxigenic *Bacteroides fragilis* (ETBF), *Fusobacterium nucleatum*, *Porphyromonas* spp., *Lactobacillus* spp., *Roseburia* spp., and *Bifidobacterium* spp. were quantified in fecal samples using absolute qRT PCR, and, finally, the association between TLRs and these gut microbiota- was evaluated by Spearman’s correlation coefficient. Higher expression of TLR2 and TLR4 in VP/TVP and TA, and lower expression levels of TLR3 and TLR5 in all type of polyps were observed. The differences in TLR expression patterns was not only dependent on the histology, location, size, and dysplasia grade of polyps but also related to the intestinal microbiota patterns. TLR2 and TLR4 expression was directly associated with the *F. nucleatum, E. faecalis*, *S. bovis*, *Porphyromonas*, and inversely to *Bifidobacterium*, *Lactobacillus*, and *Roseburia* quantity. Furthermore, TLR3 and TLR5 expression was directly associated with *Bifidobacterium*, *Roseburia*, and *Lactobacillus* quantity. Our results suggest a possible critical role of TLRs during colorectal polyp progression. An abnormal regulation of TLRs in relation to gut microbial quantity may contribute to carcinogenesis.

## 1. Introduction

Colorectal cancer (CRC) is one of the most common cancers, and accounts for almost half a million deaths annually worldwide [1,2]. CRC represents a heterogeneous group of cancers arising from at least two precursors, the conventional adenoma (CA) and the serrated polyp [3,4]. The majority of CRC cases (~60%) arise via the conventional pathway, with ~20% arising from the serrated pathway and ~20% from an alternate pathway [5]. These distinct molecular pathways dictate the different precursor lesions, such as the conventional pathway resulting in CA and the serrated pathway with sessile serrated adenomas (SSA) [4,5]. An additional serrated polyp type, the hyperplastic polyp (HP), has negligible malignant potential [6,7]. Previous evidence suggests that gut bacteria may be a major factor involved in colon cancer development [8], although distinct contributions through CAs or SSAs have not been studied simultaneously. Gut microbiota may play critical role in the progression of CRC via their metabolite or their structural components which interact with pathogen-associated molecular patterns (PAMP) and microbe-associated molecular pattern (MAMP) receptors such as Toll-like receptors (TLRs) [9,10]. TLRs are key molecules involved in inflammation and also contribute to carcinogenesis [11,12,13]. In addition to cancer development, TLRs are specifically involved in transduction of molecular signals guiding immune processes such as induction and regulation of both innate and adaptive immunity, production of cytokines, and recognition of specific molecular patterns on the surface of microorganisms [14,15,16]. In spite of the vast body of research surrounding TLRs, there is a lack of evidence on differentiating levels of TLR expression in different types of colorectal polyps as precursors of CRC [15,17].

The aim of our study was to evaluate the expression levels of TLR2, TLR3, TLR4, and TLR5 in intestinal biopsy specimens of patients with different histological colorectal polyp types including HP, SSA, tubular adenoma (TA), and villous/tubulovillous (VP/TVP) cases compared to normal controls. In addition, selected intestinal bacteria in matched fecal specimens from these participants were quantitatively analyzed to determine the association between TLRs and gut microbial patterns that may suggest a possible role of crosstalk between specific bacteria and TLRs in the progression of colon polyps to CRC.

## 2. Results

### 2.1. Characteristics of Study Groups

Demographic and clinical variables were evaluated between different categories of study groups. Participants’ characteristics with related P-value in normal, HP, SSA, VP/TVP, and TA types of polyps are presented in Table 1. Fortunately, the population studied was characterized by a similar distribution of age, gender, diabetes history, physical activity, GI disease history, alcohol consumption, and tumor location.

### 2.2. Expression of TLRs in HP, SSA, TA, VP/TVP, and Normal Controls

**Figure 1:**
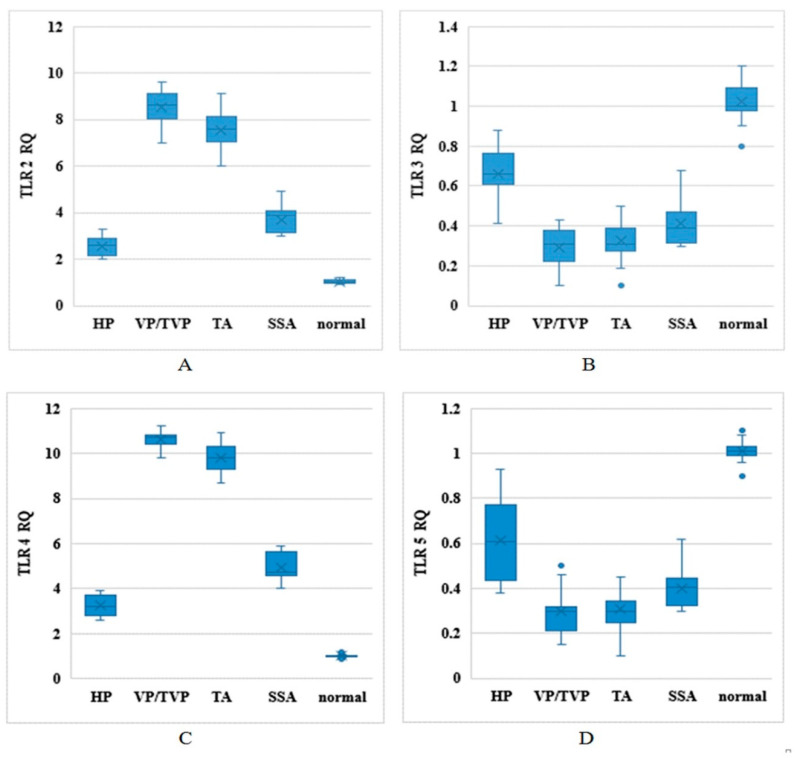
Toll-like receptor (TLR) mRNA expression levels based on relative expression (RQ) in different types of histological polyps: Distribution of TLR2, TLR3, TLR4, and TLR5 mRNA expression levels between normal, hyperplastic (HP), sessile serrated adenoma (SSA), villous/tubulovillous (VP/TVP), and tubular adenoma (TA) polyps in horizontal axis, in vertical axis; (**A**) TLR2 RQ, (**B**) TLR3 RQ, (**C**) TLR4 RQ, and (**D**) TLR5 RQ.

TLR2, TLR3, TLR4, and TLR5 mRNA expression levels were evaluated in clinical biospecimens of normal, HP, SSA, VP/TPV, and TA groups by relative qPCR and based on relative expression (RQ) levels, where normal tissue expression was set as the reference after all signals were normalized to β–2-microglobulin (Figure 1). Higher expression levels of TLR2 and TLR4 were detected in VP/TVP and TA compared to normal, HP, and SSA groups (*p*-value < 0.001). In addition, lower expression rates were observed for TLR3 and TLR5 in all polyp groups in contrast to normal individuals (*p*-value < 0.001). The noteworthy point was that the RQs of TLR2, TLR3, TLR4, and TLR5 were significantly different among histologically different colorectal polyp types compared to samples from normal participants (*p*-value < 0.001) (Table 2).

We further classified colorectal polyps by location into proximal (*n* = 33) and distal (*n* = 54), by size either <0.5 mm (*n* = 51) or ≥0.5 mm (*n* = 35), as well as having low dysplasia (*n* = 33) or medium/high dysplasia (*n* = 54). The expression of targeted TLRs was then analyzed according to the polyp characterization described above and outlined in Table 3. A significant association between TLR2 and TLR4 expression levels and location of colorectal polyps was observed. The significant relationship between the polyp size and the RQ of TLR2, 3, 4, and, 5 was demonstrated. Finally, significant associations between the RQ of TLR2, TLR4, and TLR5 and grade of dysplasia were achieved.

### 2.3. Bacterial Diversity across Different Types of Colon Polyps

**Figure 2:**
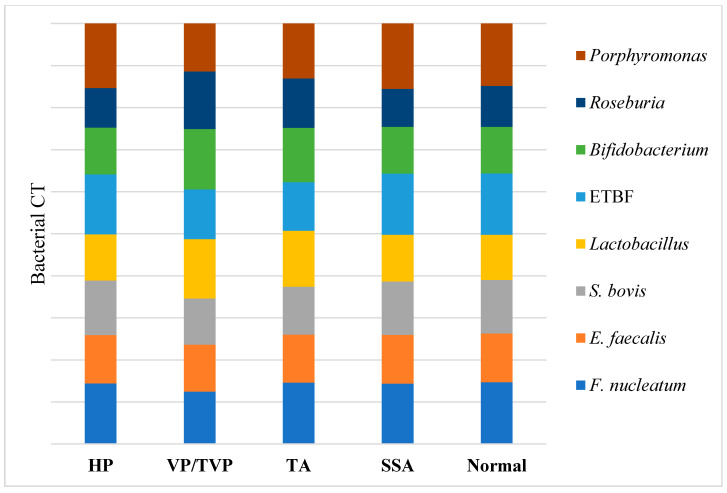
Distribution of targeted bacterial markers between hyperplastic polyp (HP), villous/tubulovillous (VP/TVP), tubular adenoma (TA), sessile serrated adenoma (SSA), and normal groups were depicted in horizontal axis. In addition, bacterial CT of *Porphyromonas* spp., *Roseburia* spp., *Bifidobacterium* spp., Enterotoxigenic *Bacteroides fragilis* (ETBF), *Lactobacillus* spp., *Streptococcus bovis*, *Enterococcus faecalis*, and *Fusobacterium nucleatum* were demonstrated in vertical axis. Higher amounts of *F. nucleatum*, *E. faecalis*, *S. bovis*, ETBF, and *Porphyromonas* spp. based on CT analysis were detected in patients’ samples with TAs and VP/TVPs (*p*-value < 0.001), while higher abundance of *Lactobacillus*, *Roseburia*, and *Bifidobacterium* were reported in normal, HP, and SSA (*p*-value < 0.001).

According to the results visualized in Figure 2 and Table S1, higher quantities of *Fusobacterium nucleatum*, *Enterococcus faecalis*, *Streptococcus bovis/gallolyticus*, Enterotoxigenic *Bacteroides fragilis*, and *Porphyromonas* spp. were detected in patients’ samples with TAs and VP/TVPs, compared to the normal fecal samples from subjects that were controls and patients with HPs and SSAs (*p*-value < 0.001). An opposite proportion was observed for the quantification of *Lactobacillus* spp., *Roseburia* spp., and *Bifidobacterium* spp., where greater numbers of bacteria were detected in normal, HP, and SSA polyp groups, compared to the TA and VP/TVP cases (*p*-value < 0.001).

Lastly, we analyzed the association between the quantity of these targeted gut bacteria (based on their real-time CT) and the RQ of the main TLRs being studied (TLR2, TLR3, TLR4, and TLR5). Table 4 shows a significant correlation between all selected bacteria (*S. bovis*, *E. faecalis*, ETBF, *F. nucleatum*, *Porphyromonas* spp., *Lactobacillus* spp., *Roseburia* spp., and *Bifidobacterium* spp.) and expression levels of all TLRs. We found that the TLR2 and TLR4 expression rates were directly associated with the quantity of *F. nucleatum*, *E. faecalis*, *S. bovis*, and *Porphyromonas* spp., but inversely related to the *Bifidobacterium*, *Lactobacillus*, and *Roseburia* spp. quantity. In addition, we observed that TLR3 and TLR5 expression rates were directly associated with *Bifidobacterium* spp., *Roseburia* spp., and *Lactobacillus* spp. quantity.

## 3. Discussion

This study focused on TLR expression in different histological types of colorectal polyps and their relationship with some selected gut bacteria. We found differences in TLR expression patterns depending on the histological type of polyps, their size, location, and grade of dysplasia, and further differentiated polyp types by fecal bacterial quantity. To the best of our knowledge, there have been limited studies investigating the expression of TLRs across different types of polyps as early precursors of CRC [18,19]. We observed significant differential mRNA expression of intestinal TLR2, 3, 4, and 5 in patents with TA, VP/TVP, HP, and SSA compared to normal individuals. In fact, TLR3 and TLR5 expression levels were higher in normal individuals, compared to different types of progressive colorectal polyps. These TLRs (3 and 5) might be essential for maintaining intestinal balance and homeostasis, suggesting a possible protective role against malignant transformation of the colorectal mucosa. In our study, TLR2 and TLR4 overexpression was observed in subjects with TA and VP/TVP polyps. Our findings that TLR3 and TLR5 are constitutively expressed in the healthy gut, while TLR2 and TLR4 appear to be overexpressed in unhealthy gut, confirmed earlier observations in studies by Yang et al., Kelly et al., and Kutikhin et al. [20,21,22]. Moossavi et al. [17] declared more evidence is required to fully determine the effect of TLRs on intestinal tumorigenesis before being able to translate and generalize the results to clinical practice. For instance, based on our previous study, we observed that aberrant surface expression of TLR9 on tumor cells may promote the growth and invasion of colorectal polyps [18], while Gao et al. [23] indicated that TLR9 signaling activation participated in the clinical process of CRC and influenced NF-kappaB expression. Bednarczyk et al. further observed a correlation in the expression levels of TLR7 and TLR9 with colorectal polyp progression to CRC [15]. These study differences could be explained by the limitations of the methods employed and lack of attention to the spatiotemporal variation in TLR expression patterns [17]. We also found that intestinal TLR2, 3, 4, and 5 expression levels were associated with location, size, and dysplasia grade of different types of colorectal polyps. In fact, our results indicated intestinal TLR expression levels may play important roles in the development of site-specific histological types of colorectal polyps.

Moreover, we have investigated the relationship between several fecal bacteria, including *F. nucleatum*, *E. faecalis*, *S. bovis*, *Lactobacillus*, ETBF, *Bifidobacterium*, *Roseburia*, and *Porphyromonas*, and intestinal TLRs and observed significant association between TLR mRNA expression levels and select microbial species abundance. An overpopulation of stool bacteria, consisting of *F. nucleatum*, *E. faecalis*, *S. bovis*, and *Porphyromonas* in relation to TLR2 and TLR4 expression level was shown in TA and VP/TVP cases, while *Roseburia* spp., *Lactobacillus* spp., and *Bifidobacterium* spp. were decreased in these cases. These results are strongly consistent with previous studies that demonstrated the interactions between gut microbiota and TLRs that impact homeostasis and immune responses or gut microbiota associated with immunological regulation and inflammation [24,25]. In fact, commensal microbiota and their structural components and products such as LPS, flagella, and DNA or RNA can be recognized by TLRs and these interactions trigger responses that help to maintain the homeostasis of intestinal immunity [26]. Overall, these findings confirm the potential utility of TLR expression patterns in relation to gut microbiota for discriminating polyp cases from normal group samples, and suggest that changes in microbial abundance in combination with specific TLR relative levels may represent early events in the pathway(s) leading to colorectal polyp. Although our results are promising, a limitation of our study is that we only profiled eight selected bacterial species, which may not represent the full biodiversity within our patient population. Future studies will take a more comprehensive approach to microbial profiling as well as seek to correlate both this biodiversity with a specific set of inflammation markers regulated through NF-kappaB.

In conclusion, the present study has shown significant difference in the expression levels of intestinal TLR2, 3, 4, and 5, in patients with TA, VP/TVP, HP, and SSA polyps versus healthy controls. Based on current findings, TLR2 and TLR4 may be upregulated in the process of polyp formation and progression. In contrast, TLR3 and TLR5 were downregulated in all patient polyp types compared with normal tissue samples. Hence, they might be essential for maintaining balance and homeostasis in a healthy gut and have a possible protective role against malignant transformation of the colorectal mucosa. In addition, we found that intestinal TLR2, 3, 4, and 5 expression levels associate with location, size, and dysplasia grade of colorectal polyps and their expression levels may play important roles in the development of site-specific histological types of colorectal polyps. Moreover, there was a clear correlation between TLR mRNA expression levels and specific fecal bacterial species, suggesting an abnormal activation or regulation of TLRs in relation to gut microbiota which may promote disease progression. Finally, more evidence is required to fully appreciate the effect of differential TLR expression on intestinal tumorigenesis, and data on larger cohorts are necessary to validate and translate the results into clinical practice. Further studies on TLR expression patterns and gut microbial interactions in colorectal polyps can improve strategies in CRC prevention and earlier detection.

## 4. Materials and Methods

### 4.1. Sample Collection and Storage

One-hundred and eighteen colonic biopsy and fecal specimens, including samples from 31 normal controls, 21 HP, 16 SSA, 29 TA, and 21 VP/TVP, were collected from candidate individuals at Taleghani Hospital, Tehran, Iran between 2016 and 2018. Eligible participants were individuals 50–80 years old and scheduled to have a colonoscopy for routine screening. Additional demographic data characterizing the study cohort are summarized in Table 1. Colonic tissue samples included in the study were consented from these participants at the time of colonoscopy and provided during the procedure. All fresh stool samples were collected three days to two weeks before colonoscopy and bowel cleansing procedures associated with the routine screen [27,28]. All colonic biopsy samples were classified after colonoscopy and confirmed by an expert pathologist. Fecal and biopsy samples were stored at −80 °C immediately until further analysis. The case-control study was approved by the Clinical Research Ethics Committee of the Shahid Beheshti University of Medical Sciences and the Ethics Committee of Taleghani Hospital, Tehran, Iran (No. 851; 2016-12-7). 

### 4.2. RNA Extraction from Colon-Derived Tissues

RNA was extracted from the colonic biopsy and normal tissues using the TRIzol reagent (ThermoFisher Scientific, Massachusetts, United States) in accordance with the manufacturer’s guidelines. After final precipitation, the DNA was resuspended in Tris-EDTA (TE) buffer and stored at −20 °C for further analysis.

### 4.3. DNA Extraction from Fecal Specimens

Genomic DNA was extracted from frozen preserved fecal samples using the QIAamp DNA Stool Mini Kit (QIAGEN, Hilden, Germany) in accordance with the manufacturer’s instructions.

### 4.4. TLR mRNA Expression Levels in Extracted RNA from Colonic Tissues

TLR2, TLR3, TLR4, and TLR5 mRNA expression levels were evaluated in extracted RNA from tissue samples using the Premix Ex Taq SYBR (Takara Bio, Kusatsu, Japan) and the relative qRT-PCR technique in accordance with the manufacturer’s guidelines. Specific primers were selected from previously published studies [29,30,31]. Amplification signals for samples were normalized by β–2-microglobulin [32] and relative fold change of TLRs genes expression was evaluated by the 2^−ΔΔ*C*T^ method [18,32].

### 4.5. Quantification of Bacterial Species by 16SrRNA in Extracted DNA from Fecal Samples

Bacterial 16SrRNA quantification for several target gut bacteria including *S. bovis*, *E. faecalis*, ETBF, *F. nucleatum*, *Porphyromonas* spp., *Lactobacillus* spp., *Roseburia* spp., and *Bifidobacterium* spp. was precisely determined in fecal samples by absolute qRT PCR using the SYBR Green detection system, as described in our previous study [27]. The association between mRNA expression levels of TLR2, TLR3, TLR4, and TLR5 and the quantification of the selected gut microbiota was evaluated by statistical analysis and Spearman’s correlation coefficient.

### 4.6. Statistical Analysis

The descriptive statistics were expressed for numerical and categorical variables using mean (standard deviation (SD)/median (interquartile range (IQR)) and frequency (percentage), respectively. The association between categorical variables and polyp groups was assessed by Pearson chi-square and Fisher exact test. In addition, analysis of variance (ANOVA) was used to compare the mean numerical variables (including demographic and TLRs) between polyp types. The assumption of normality was checked for all numeric variables and the non-parametric test were used when the data were not normally distributed. Accordingly, the distribution of tumor size was compared between polyp types using the Kruskal–Wallis test. The Mann–Whitney U test was used to compare the difference in TLRs between each level of polyp location, polyp size, and grade of dysplasia. The association between TLRs and candidate bacteria was evaluated using Spearman’s correlation coefficient. A box plot and bar plot were used to indicate the distribution of TLR mRNA expression levels based on RQ and bacterial species in different colon polyp types. All analyses were performed in R (version 4.02) and SPSS (version 26) and a *p*-value of less than 0.05 was regarded as statistically significant.

## Figures and Tables

**Table 1:** Patient demographics in normal, hyperplastic polyp (HP), sessile serrated adenoma (SSA), tubular adenoma (TA), and villous/tubulovillous (VP/TVP) groups.

Variable	Normal	HP	SSA	VP/TVP	TA	*p*-Value
N (%)	31 (26.3)	21 (17.8)	16 (13.6)	21 (17.8)	29 (24.6)	----
Age, mean (SD)	59.84 (17.0)	60.19 (15.2)	62.25 (14.3)	57.33 (12.7)	58.03 (12.7)	0.849 ^a^
Gender, N (%)						0.242 ^b^
Female	14 (45.2)	5 (23.8)	4 (25.0)	10 (47.6)	14 (48.3)	
Male	17 (54.8)	16 (76.2)	12 (75.0)	11 (52.4)	15 (51.7)	
Family history, N (%)						<0.001 ^c,^**
No	31 (100.0)	20 (95.2)	16 (100.0)	13 (61.9)	22 (75.9)	
Yes	0 (0.0)	1 (4.8)	0 (0.0)	8 (38.1)	7 (24.1)	
Diabetes mellitus history, N (%)						0.657 ^c^
No	29 (93.5)	18 (85.7)	14 (87.5)	17 (81.0)	24 (82.8)	
Yes	2 (6.5)	3 (14.3)	2 (12.5)	4 (19.0)	5 (17.2)	
Smoking, N (%)						0.006 ^c,^*
No	24 (77.4)	13 (61.9)	10 (62.5)	21 (100.0)	25 (86.2)	
Yes	7 (22.6)	8 (38.1)	6 (37.5)	0 (0.0.)	4 (13.8)	
Physical activity, N (%)						0.374 ^b^
Low	21 (67.7)	13 (61.9)	8 (50.0)	17 (81.0)	20 (69.0)	
High	10 (32.3)	8 (38.1)	8 (50.0)	4 (19.0)	9 (31.0)	
GI diseases history, N (%)						0.249 ^c^
No	7 (22.6)	3 (14.3)	2 (12.5)	8 (38.1)	4 (13.8)	
Yes	24 (77.4)	18 (85.7)	14 (87.5)	13 (61.9)	25 (87.5)	
Alcohol, N (%)						0.128 ^c^
No	30 (96.8)	19 (92.5)	14 (87.5)	21 (100.0)	29 (100.0)	
Yes	1 (3.2)	2 (9.5)	2 (9.5)	0 (0.0)	0 (0.0)	
Tumor location, N (%)						0.157 ^b^
Proximal	0 (0.0)	6 (28.6)	4 (25.0)	12 (57.1)	11 (37.9)	
Distal	0 (0.0)	5 (71.4)	12 (75.0)	9 (42.9)	18 (62.1)	
Grade, N (%)						<0.001 ^b,^**
Low (= 0)	0 (0.0)	17 (81.0)	12 (75.0)	0 (0.0)	4 (13.8)	
Medium/high (≥1)	0 (0.0)	4 (19.0%)	4 (25.0)	21 (100.0)	25 (86.2)	
Fecal occult blood test, N (%)						0.008 ^c,^*
No	31 (100.0)	21 (100.0)	16 (100.0)	17 (81.0)	25 (86.2)	
Yes	0 (0.0)	0 (0.0)	0 (0.0)	4 (19.0)	4 (13.8)	
Tumor size, median (IQR)	---	4.0 (3.0–4.0)	4.0 (3.3–5.0)	6.0 (4.0–10.5)	4.0 (3.0–5.0)	0.002 ^d,^**

^a^ Analysis of variance for comparing the mean variables in different groups of polyps, ^b^ the chi-square test for testing the association between categorical variable and different groups of polyps, ^c^ the Fisher exact test for evaluation of the association between categorical variable and different groups of polyps, ^d^ the Kruskal–Wallis test for comparing the mean rank of variables between different groups of polyps, * test is significant at the 0.05 level (2-tailed), ** test is significant at the 0.01 level (2-tailed).

**Table 2:** Expression of candidate TLRs in normal, HP, SSA, VP/TPV, and TA groups.

Group	Normal, *n* = 31	HP, *n* = 21	SSA, *n* = 16	VP/TVP, *n* = 21	TA, *n* = 29	*p*-Value
TLR2 (RQ)	1.03 (0.08) ^a^	2.56 (0.44)	3.70 (0.56)	8.52 (0.73)	7.56 (0.86)	<0.001 *
TLR3 (RQ)	1.03 (0.09)	0.66 (0.13)	0.41 (0.11)	0.29 (0.10)	0.32 (0.09)	<0.001 *
TLR4 (RQ)	1.01 (0.06)	3.24 (0.45)	4.91 (0.64)	10.60 (0.37)	9.81 (0.65)	<0.001 *
TLR5 (RQ)	1.01 (0.06)	0.61 (0.18)	0.40 (0.09)	0.30 (0.09)	0.31 (0.10)	<0.001 *

^a^ Mean (SD) of mRNA expression is based on mean of relative expression (RQ). Toll-like receptors (TLRs), relative expression (RQ), hyperplastic (HP), sessile serrated adenoma (SSA), villous/tubulovillous (VP/TVP), and tubular adenoma (TA). * The mean of TLRs was significantly different at the level of 0.01 using analysis of variance after adjusting for family history and smoking status.

**Table 3:** Association between the relative expression of TLR2, 3, 4, and 5 and polyp size, location, and grade of dysplasia.

Group	Polyp Size (mm)			Dysplasia Grade			Polyp Location		
	<0/5 *n* = 51	≥/5 *n* = 35	*p*-Value *	Low *n* = 33	Medium & High *n* = 54	*p*-Value *	Distal *n* = 54	Proximal *n* = 33	*p*-Value *
TLR2 (RQ)	3.59 (1.79)	7.27 (1.93)	<0.001	5.07 (2.48)	7.13 (2.24)	<0.001	5.42 (2.53)	6.61 (2.55)	0.034
TLR3 (RQ)	0.53 (0.18)	0.34 (0.14)	<0.001	0.45 (0.19)	0.35 (0.15)	0.014	0.43 (0.18)	0.38 (0.18)	0.167
TLR4 (RQ)	4.69 (2.25)	9.24 (2.31)	<0.001	6.60 (3.24)	8.99 (2.43)	0.001	6.93 (3.17)	8.47 (3.01)	0.007
TLR5 (RQ)	0.51 (0.19)	0.33 (0.12)	<0.001	0.44 (0.20)	0.32 (0.10)	0.002	0.40 (0.17)	0.39 (0.18)	0.782

* The Mann–Whitney U test for comparing the differences between distal and proximal. Toll-like receptors (TLRs), relative expression (RQ).

**Table 4:** Association between candidate bacteria and the relative expression of TLR2, TLR3, TLR4, and TLR5.

Group	Candidate Bacteria							
	*F. nucleatum* CT	*E. faecalis* CT	*S. bovis* CT	*Lactobacillus* CT	ETBF CT	*Bifidobacterium* CT	*Roseburia* CT	*Porphyromonas* CT
TLR2 (RQ)	−0.61 **	−0.48 **	−0.73 **	0.61 **	−0.79 **	0.46 **	0.43 **	−0.69 **
TLR3 (RQ)	0.46 **	0.43 **	0.60 **	−0.57 **	0.68 **	−0.36 **	−0.36 **	0.60 **
TLR4 (RQ)	−0.58 **	−0.46 **	−0.74 **	0.61 **	−0.75 **	0.48 **	0.45 **	−0.70 **
TLR5 (RQ)	0.47 **	0.40 **	0.61 **	−0.63 **	0.69 **	−0.37 **	−0.35 **	0.57 **

** The correlation analyzed by Spearman’s correlation is significant at the 0.01 level (2-tailed).

## Supplementary Materials

The following are available online at https://www.mdpi.com/1422-0067/21/23/8968/s1.

## Abbreviations

TLRs	Toll-like receptors
HP	hyperplastic polyp
SSA	sessile serrated adenoma
TA	tubular adenoma
VP/TVP	villous/tubulovillous polyp
ETBF	Enterotoxigenic *Bacteroides fragilis*
CRC	colorectal cancer
RQ	relative expression
