# Supplementary material for: Expression of Main Toll-Like Receptors in Patients with Different Types of Colorectal Polyps and Their Relationship with Gut Microbiota
Source: Int J Mol Sci. 2020 Nov 26;21(23):8968. doi: 10.3390/ijms21238968 (PMC7729598; doi:10.3390/ijms21238968)
Supplement: Supplementary file 1 [file ijms-21-08968-s001.pdf]

International Journal of *Molecular Sciences*

MDPI

Table S1. Distribution of *F.nucleatum, E. faecalis, S.bovis, Lactobacillus* spp., ETBF*, Bifidobacterium* spp., *Roseburia* spp., and *Porphyromonas* spp. based on CT in normal, hyperplastic (HP), sessile serrated adenoma (SSA), villous/ tubulovillous (VP/TVP), and tubular adenoma (TA) polyp groups.

**Bacteria**	**Normal,** ***n*** **= 31**	**HP,** ***n*** **= 21**	**SSA,** ***n*** **= 16**	**VP/TVP,** ***n*** **= 21**	**TA,** ***n*** **= 29**	
F.nucleatum CT	29.42 (2.94)	28.37 (1.94)	27.98 (2)	21.41 (3.48)	26.46 (2.67)	<0.001
E. faecalis CT	23.12 (1.36)	22.6 (2.15)	22.63 (1.94)	19.28 (2.66)	20.64 (2.85)	<0.001
S.bovis CT	25.48 (2.31)	25.49 (2.11)	24.74 (1.84)	18.84 (2.65)	20.62 (1.76)	<0.001
Lactobacillus spp. CT	21.48 (1.56)	21.62 (1.61)	21.65 (1.58)	24.27 (1.46)	24.09 (1.37)	<0.001
ETBF CT	29.16 (2.65)	28.07 (2.21)	28.4 (1.5)	20.4 (1.54)	20.96 (2.02)	<0.001
Bifidobacterium spp. CT	22.13 (1.51)	21.86 (1.31)	21.71 (1.29)	24.62 (3.38)	23.43 (1.51)	<0.001
Roseburia spp. CT	19.42 (3.55)	18.51 (3.4)	17.59 (2.38)	23.58 (1.9)	21.26 (2.15)	<0.001
Porphyromonas spp. CT	29.62 (2.23)	30.13 (2.15)	30.23 (2.2)	19.57 (2.61)	23.59 (3.65)	<0.001
